# Effect of Colonoscopy Volume on Quality Indicators

**DOI:** 10.1155/2016/2580894

**Published:** 2016-05-12

**Authors:** David Pace, Mark Borgaonkar, Muna Lougheed, Curtis Marcoux, Brad Evans, Nikita Hickey, Meghan O'Leary, Darrell Boone, Jerry McGrath

**Affiliations:** Department of Surgery, Memorial University, St. John's, NL, Canada A1B 3V6

## Abstract

*Background*. The purpose of this study is to determine if colonoscopy quality is associated with the annual case volume of endoscopists.* Methods*. A retrospective cohort study was performed on 3235 patients who underwent colonoscopy in the city of St. John's, NL, between January and June 2012. Data collected included completion of colonoscopy (CCR) and adenoma detection rates (ADR). Endoscopists were divided into quintiles based on annual case volume. To account for potential confounding variables, univariate analyses followed by multivariable logistic regression were used to identify variables independently associated with CCR and ADR.* Results*. A total of 13 surgeons and 8 gastroenterologists were studied. There was a significant difference in CCR (*p* < 0.001) and ADR (*p* < 0.001) based on annual volume. Following multivariable regression, predictors of successful colonoscopy completion included annual colonoscopy volume, lower age, male sex, an indication of screening or surveillance, and a low ASA score. Predictors of adenoma detection included older age, male sex, an indication of screening or surveillance, and gastroenterology specialty.* Conclusion*. Higher annual case volume is associated with better quality of colonoscopy in terms of completion. However, gastroenterology specialty appears to be a better predictor of ADR than annual case volume.

## 1. Introduction

The National Polyp Study suggested that colonoscopy with polypectomy can reduce the chance of subsequent colorectal cancer by as much as 90% [1]. More recent studies have found less of an impact [2–4] and at least one study has suggested that this benefit is only limited to the left colon [5]. Some of these differences are likely the result of missed adenomas and incomplete colonoscopy. Thus, ensuring high-quality colonoscopy is a goal to which all endoscopists should aspire.

There has been some research on how many colonoscopies need to be performed annually to maintain high-quality outcomes. The Society of Gastrointestinal and Endoscopic Surgeons (SAGES) Colonoscopy Outcomes Study Group found in 2001 that endoscopists were significantly more likely to complete colonoscopy to the cecum if they had performed at least 100 colonoscopies annually [6]. Another recent study found a similar relationship between colonoscopy volume and cecal intubation [7].

Despite numerous proposed quality indicators for colonoscopy, few measures have been validated. For our study, we chose quality measures that have been shown to translate into improved screening efficacy. A recent large-scale population based study demonstrated that both higher completion rates and polyp detection rates are independently associated with a reduced risk of postcolonoscopy colorectal cancer [8]. Further studies have demonstrated an independent association between adenoma detection rate and risk of colorectal cancer following screening colonoscopy [9, 10]. Accordingly, we report colonoscopy completion rate (CCR), polyp detection rate (PDR), and adenoma detection rate (ADR) as measures of quality colonoscopy.

The purpose of this study is to determine if colonoscopy quality, as measured by colonoscopy completion, polyp detection, and adenoma detection, is associated with the annual case volume of endoscopists. We also sought any other factors that may predict these quality measures.

## 2. Methods

### 2.1. Data Collection

A retrospective cohort study was performed on 3235 patients who underwent colonoscopy in the city of St. John's, NL, between January and June 2012. Data were obtained from the endoscopy procedure reports, the nursing records of the endoscopy, and the pathology reports in the electronic medical record system. Data on a number of variables including colonoscopy completion, adenoma detection, polyp detection, perforation, and postpolypectomy bleeding rates were collected. Colonoscopy completion was based on endoscopist opinion, as photo documentation of completion was inconsistently performed. Adenomas were defined as any of adenomas, serrated adenomas, or malignant polyps in the pathology report.

Endoscopists were divided into quintiles based on annual case volume (1st, 0–149; 2nd, 150–249; 3rd, 250–301; 4th, 302–530; 5th, >530). Endoscopists were placed into these quintiles based on the number of colonoscopies they performed over a two-year period, from January 2011 to December 2012. Quality outcomes were calculated based upon these quintiles.

Potential confounding variables that were considered included patient level variables (patient age, gender, indication for colonoscopy, and ASA score (1 or 2 versus 3 to 5)) and an endoscopist-level variable (specialty, general surgery versus gastroenterology). The indications for colonoscopy were divided into two groups: colonoscopies performed for colorectal cancer screening and surveillance purposes excluding inflammatory bowel disease or colonoscopies performed for other reasons including patient symptoms, anemia, abnormal findings on diagnostic imaging, and inflammatory bowel disease.

Quality of bowel preparation was not used in the analysis because it was only recorded in 20% of the endoscopy reports and was not reported in a standardized manner.

The study was granted approval by the local ethics committee.

### 2.2. Colonoscopy Information

A total of 13 surgeons and 8 gastroenterologists were studied. All procedures were performed using either adult (EC-530HL) or paediatric (EC-530LS) colonoscopes made by the Fujinon Corporation (Fujinon Corporation, Saitama City, Japan). Air insufflation was used for distending the colon. The sedation consisted of intravenous midazolam and/or fentanyl. The bowel preparation used was at the discretion of the endoscopist. The preparations used consisted mainly of either a polyethylene glycol based preparation or a sodium picosulfate based preparation. Split-dose preparations were not used by any of the endoscopists during the study period.

### 2.3. Outcome Definitions

The CCR was calculated as the proportion of all colonoscopies in which the endoscopist reached the cecum, anastomosis, or terminal ileum. The ADR was calculated as the proportion of colonoscopies in which one or more histologically confirmed adenomas were found. The PDR was calculated as the proportion of colonoscopies in which one or more polyps were found, regardless of histologic type. The perforation rate was calculated as the proportion of colonoscopies in which a bowel perforation was suspected clinically to have occurred. The postpolypectomy bleeding rate was calculated as the proportion of all colonoscopies in which a patient returned to hospital with rectal bleeding within 14 days of a polypectomy.

### 2.4. Statistical Analysis

SPSS version 19.0 was used for analysis (SPSS, Chicago, Illinois, USA). Student's *t*-test and analysis of variance were used for continuous variables and chi-squared test for categorical variables.

To account for potential confounding variables, univariate analysis was performed to identify variables associated with colonoscopy completion (*p* < 0.10), polyp detection (*p* < 0.10), and adenoma detection (*p* < 0.10). Multivariable step-wise logistic regression was used to identify variables independently associated with the outcomes of interest (*p* < 0.05). An odds ratio (OR) and a 95% confidence interval were created for each independent variable. An odds ratio exceeding 1.00 indicated an increased likelihood of an event occurring for a risk variable (such as male gender when analysing gender) compared with a reference category (female). Conversely, an odds ratio less than 1.00 indicated a decreased likelihood of an event occurring to the risk variable (such as the first-volume quintile when analysing volume) compared with the reference category (the fifth-volume quintile).

As 3 endoscopists (all general surgeons) had less than 5 years of experience, a sensitivity analysis was performed excluding their outcomes. The results of the sensitivity analysis were then compared to the main analysis that included these surgeons.

### 2.5. Power Analysis

Using CCR as the primary outcome measure, if we assume a 92% completion rate for the 3235 cases (260 failed colonoscopies) and if we allow for 15–20 events (failures) per independent variable, at least 13 independent variables could be assessed.

## 3. Results

A total of 3235 cases involving 13 general surgeons and 8 gastroenterologists were studied. Though there were 6 patients with no dictated reports from the endoscopist, the CCR, the ADR, and the PDR from these cases could be extracted from the nursing and pathology records. Median patient age was 59 years with 55.8% of the group being female. The majority of patients (97.1%) were outpatients and were ASA class 1 or 2 (85.7%). Most of the patients underwent colonoscopy for asymptomatic screening or surveillance (61.3%). General surgeons performed 36.7% of cases and gastroenterologists performed the rest. As a group, the overall CCR was 92.0%, the ADR was 21.83%, the PDR was 35.65%, the colonoscopic perforation rate was 0.20%, and the postpolypectomy bleeding rate was 0.20%. There were 19 patients (0.6%) who had unplanned hospitalization within 14 days of their colonoscopy.

The distribution of potentially confounding variables within the five different volume quintiles can be seen in Table 1.

There was a significant difference in completion of colonoscopy based on annual colonoscopy volume of the endoscopist (80.7% in the first quintile to 93.3% in the 5th quintile; *p* < 0.001). Polyp detection rates increased with higher annual case volume (25.1% in the first quintile to 40.9% in the 5th quintile; *p* < 0.001). Furthermore, adenoma detection rates were also higher for the endoscopists with the highest case volumes (16.0% in the first quintile to 23.7% in the 5th quintile; *p* < 0.001). The colonoscopic perforation rate and the postpolypectomy bleeding rate did not vary with annual volume. See Table 2.

Patients who underwent a successful colonoscopy were younger than those with an incomplete colonoscopy (mean age 58.2 versus 60.6 years; *p* = 0.002). Univariate analysis also identified male gender, a lower ASA score (1 or 2), gastroenterology specialty, and an indication of screening or surveillance as variables associated with greater colonoscopy completion. See Table 3. Using multivariable logistic regression, all of the above variables were associated with colonoscopy completion except for endoscopist specialty (*p* = 0.72). Annual colonoscopy volume of less than 250 cases was a predictor of incomplete colonoscopy relative to the reference group. See Table 4.

Patients who had an adenoma removed were older than those who did not have an adenoma removed (mean age 61.9 versus 57.4 years; *p* < 0.001). Univariate analysis also identified male gender, a higher ASA score (3 to 5), gastroenterology specialty, and an indication of screening or surveillance as variables associated with higher adenoma detection. See Table 3. Using multivariable logistic regression, all of the above variables were associated with adenoma detection except for colonoscopy volume (*p* = 0.837) and a high ASA score (*p* = 0.199). See Table 5.

Patients who had a polyp removed were older than those who did not have a polyp removed (mean age 61.2 versus 56.7 years; *p* < 0.001). Univariate analysis also identified male gender, a higher ASA score (3 to 5), gastroenterology specialty, and an indication of screening or surveillance as variables associated with higher polyp detection. See Table 3. Using multivariable logistic regression, all of the above variables were associated with polyp detection except for colonoscopy volume (*p* = 0.538). See Table 6.

The sensitivity analysis performed, which excluded the outcomes of three inexperienced endoscopists, had no effect upon the results of the univariate or multivariable logistic regression analyses.

## 4. Discussion

In this study, we found that the likelihood of completing a colonoscopy is directly related to annual colonoscopy volume. This is consistent with other studies that looked at this outcome [6, 7]. In a study by Bhangu et al., annual colonoscopy volume increased from less than 100 to greater than 200 and the cecal intubation rate increased proportionately [7]. In our study, this relationship continued on to the 4th highest quintile of endoscopists, who performed over 300 cases annually. It is interesting to note that the high volume endoscopists tended to be gastroenterologists and the low volume endoscopists were surgeons. This is due to the limited access surgeons have to the endoscopy unit in our center. In many community hospitals across Canada, general surgeons perform all flexible endoscopy procedures and their annual volumes tend to be higher.

When other potential confounding variables were taken into consideration, we found that younger age, male gender, healthier patients, patients undergoing colonoscopy for screening or surveillance purposes, and an annual colonoscopy volume of at least 250 were predictors of colonoscopy completion.

Annual colonoscopy volume also appears to be related to polyp and adenoma detection. Current literature with regard to annual colonoscopy volume and polyp and adenoma detection is limited and contradictory [7, 11]. However, when other potential confounding variables were considered, this relationship disappeared and seemed to be displaced by gastroenterology specialization in the regression model. These two variables are closely related, as the gastroenterologists in this study were also the high volume endoscopists. Other variables associated with ADR included advanced age, male gender, and an indication of screening or surveillance.

Age and gender are variables that have been well studied in the past and our study is in agreement with the literature, both in terms of CCR, ADR, and PDR [12, 13]. Also, the finding that patients undergoing colonoscopy for either screening or surveillance of colorectal cancer are more likely to have adenomas than patients undergoing colonoscopy for other reasons is supported in the literature [14].

While procedural experience is undoubtedly important in developing the technical expertise required for high-quality colonoscopy, few studies have examined the precise number of annual colonoscopies needed to maintain this skill level. We observed that performing a minimum of 250 annual colonoscopies is associated with improved completion rates. We also observed that as annual colonoscopy volume increased to over 530 cases, so did the ADR and PDR. These numbers are large compared to the annual volume of many endoscopists. In a recent British study of 29 endoscopists, the average number of colonoscopies performed annually by physicians and surgeons was 130 [7]. In the United States, a recent cross-sectional study of Medicare claims revealed that the median annual number of outpatient colonoscopies performed by physicians and surgeons was only 55 [15]. In order to achieve these improved outcomes, endoscopists may need to do more procedures annually. This may require some rationing of colonoscopy resources or, possibly, endoscopists may need to undergo further training to improve their skills. A study out of Germany found that the number of continuing medical education events attended by endoscopists directly correlated with the quality of colonoscopy as measured by ADR [11]. A recent randomised trial has shown that an upskilling colonoscopy course can lead to improvement in adenoma detection among practicing endoscopists [16]. In Canada and Britain, similar hands-on colonoscopy courses aimed at practicing endoscopists have been developed and are being used to improve quality outcomes.

Why sicker patients (i.e., those with ASA scores of 3 to 5) have lower colonoscopy completion rates than healthier patients is a matter of speculation. It could be that sicker patients are less able to tolerate a bowel preparation, leading to poorer visualization of the colon. Similarly, they may be less likely to tolerate the sedation required to complete the procedure and may have more difficult colons to navigate due to conditions like chronic constipation and diverticulosis.

In univariate analysis, sicker patients were also found to have more adenomas and polyps than healthier patients. However, in the regression model, the ASA score was no longer an independent predictor of ADR. This is likely due to the fact that the sicker patients were likely older, which was also included in the model. There is very little evidence in the literature on this topic. In their review, Singh et al. did find that a high comorbidity index, as measured by the Charlson comorbidity index, was associated with an increased risk of interval cancer detection [17].

Factors in this study that could explain the differences in ADR and PDR between gastroenterologists and general surgeons, aside from volume, include (1) differences in training and (2) differences in the cumulative experience of the two groups. The gastroenterologists in this study each undertook 2 years of fellowship training while the general surgeons received 3 months of technical training in endoscopy as part of their five- or six-year general surgery residency. All gastroenterologists in this study had been in clinical practice for a minimum of 5 years, while 3 of the 13 general surgeons had been performing colonoscopy for less than 5 years. The 3 inexperienced surgeons were also the low volume endoscopists with the lowest ADRs, PDRs, and CCRs. A sensitivity analysis was performed, excluding the inexperienced group, and the results were unchanged. Studies assessing endoscopist experience have found that endoscopists with more experience have higher ADRs [18] and are more likely to detect smaller polyps and polyps of higher histological grade [19].

The evidence to support the notion that gastroenterologists find more polyps and adenomas is conflicting. In a Polish study, Kaminski et al. [9] showed that 53.1% of gastroenterologists had an ADR of over 20%, while only 23.1% of surgeons had an ADR of over 20%. Despite this, there was no difference in the risk of interval cancer. Using US Medicare data, colonoscopy performed by a gastroenterologist was more likely to result in the removal of more polyps than providers who were not gastroenterologists [15]. Conversely, others have found that surgeons have a higher PDR [7] than gastroenterologists and an equivalent ADR [7, 20].

There are a number of limitations to this study. It is a relatively small, retrospective study that only evaluated twenty-one endoscopists. Two potential confounding variables that were not accounted for in our analysis include quality of bowel preparation and withdrawal time. Bowel preparation quality was not reported in a standardized fashion but likely did not affect the results of this study given that all endoscopists used the same standardized bowel preparations. Withdrawal time was rarely reported and could not be included in our analysis.

In conclusion, higher annual case volume is associated with improved colonoscopy completion. In contrast, gastroenterology specialty was the only endoscopist factor associated with polyp detection and adenoma detection. These results may have potential impacts upon the future training and credentialing of endoscopists.

## Figures and Tables

**Table 1:** Distribution of potential confounding variables within the five endoscopist annual colonoscopy volume quintiles.

	Quintile 1	Quintile 2	Quintile 3	Quintile 4	Quintile 5	*p* value
	0–149	150–249	250–301	302–530	>530	
*Mean age* (years)	57.8	59.5	58.9	58.9	57.4	0.014

*Gender* (% male)	46.0	50.4	41.5	39.7	46.3	0.002

*ASA score* (% 1 or 2)	86.1	94.9	87.3	81.0	85.3	<0.001

*Indication* (% screening or surveillance)	57.2	68.4	51.8	58.4	66.1	<0.001

*Ratio of general surgeons to gastroenterologists *(% surgeon)	5 : 0	4 : 0	2 : 2	2 : 2	0 : 4	<0.001
	100	100	50	50	0	

**Table 2:** Quality indicators and their relationship to annual colonoscopy volume quintiles.

	Quintile 1	Quintile 2	Quintile 3	Quintile 4	Quintile 5	*p* value
	0–149	150–249	250–301	302–530	>530	
Colonoscopy completion (%)	80.7	90.9	90.5	93.9	93.3	<0.001
Polyp detection (%)	25.1	25.7	30.4	39.5	40.9	<0.001
Adenoma detection (%)	16.0	16.1	19.5	20.1	23.7	<0.001
Perforation rate (%)	0.05	0	0.2	0.2	0.2	0.716
Postpolypectomy bleeding (%)	0	0.3	0	0.05	0.02	0.235

**Table 3:** Univariate analysis of potential confounding variables associated with colonoscopy completion, adenoma detection, and polyp detection.

	Colonoscopy completion	*p* value	Adenoma detection	*p* value	Polyp detection	*p* value
*Indication (%)*						
Screening or surveillance	93.5	<0.001	24.8	<0.001	40.2	<0.001
Other	89.5		17.7		29.2	

*ASA score (%)*						
ASA (3 to 5)	88.2	<0.001	29.7	<0.001	48.6	<0.001
ASA (1 or 2)	92.7		21.2		34.0	

*Mean patient age (years)*						
Yes task completed	60.6	<0.001	61.9	<0.001	61.2	<0.001
No task incomplete	58.2		57.4		56.7	

*Gender (%)*						
Female	90.2	<0.001	17.8	<0.001	31.5	<0.001
Male	94.1		27.3		41.7	

*Specialty (%)*						
Gastroenterology	93.1	<0.001	25.0	<0.001	40.1	<0.001
General surgery	90.1		16.9		28.8	

**Table 4:** Multivariable logistic regression model for colonoscopy completion rate.

	Odds ratio	95% confidence interval	*p* value
*Indication*			
Screening or surveillance	Reference	0.457–0.808	0.001
Other	0.608		

*ASA score*			
ASA (3 to 5)	Reference	1.105–2.321	0.013
ASA (1 or 2)	1.601		

*Age*	0.984	0.972–0.996	0.007

*Gender*			
Female	Reference	1.345–2.453	<0.001
Male	1.817		

*Volume*			
Quintile 1 (0–149)	0.200	0.112–0.356	<0.001
Quintile 2 (150–249)	0.648	0.421–0.997	0.048
Quintile 3 (250–301)	0.699	0.467–1.045	0.081
Quintile 4 (302–530)	1.239	0.846–1.815	0.272
Quintile 5 (>530)	Reference		<0.001

**Table 5:** Multivariable logistic regression model for adenoma detection.

	Odds ratio	95% confidence interval	*p* value
*Indication*			
Screening or surveillance	Reference	0.590–0.849	<0.001
Other	0.708		

*Age*	1.033	1.025–1.040	<0.001

*Gender*			
Female	Reference	1.480–2.086	<0.001
Male	1.757		

*Specialty*			
General surgery	Reference	1.475–2.145	<0.001
Gastroenterology	1.779		

**Table 6:** Multivariable logistic regression model for polyp detection.

	Odds ratio	95% confidence interval	*p* value
*Indication*			
Screening or surveillance	Reference	0.501–0.709	<0.001
Other	0.596		

*ASA score*			
ASA (3 to 5)	Reference	0.558–0.888	<0.001
ASA (1 or 2)	0.704		

*Age*	1.033	1.026–1.041	<0.001

*Gender*			
Female	Reference	1.369–1.897	<0.001
Male	1.612		

*Specialty*			
General surgery	Reference	1.792–2.665	<0.001
Gastroenterology	2.185		
